# The effect of adding physician recommendation in digitally-enabled outreach for COVID-19 vaccination in socially/economically disadvantaged populations

**DOI:** 10.1186/s12889-024-18648-x

**Published:** 2024-07-18

**Authors:** Kamal Sumar, Lisa Blue, Gina Fatahi, Mehek Sumar, Stephanie Alvarez, Pedro Cons, Nathalie Valencia, Zachary Williams, Atiq Bhatti, Sairam Parthasarathy, Chyke A. Doubeni

**Affiliations:** 1 Adelante Healthcare, Phoenix, AZ USA; 2 Providertech LLC, Phoenix, AZ USA; 3 Department of Family and Community Medicine, Ohio State University, Columbus, OH USA; 4 Division of Biological Sciences, University of California San Diego, San Diego, CA USA; 5 Department of Neurologic Surgery, Mayo Clinic, Rochester, MN USA; 6 College of Medicine, University of Arizona, Tucson, AZ USA

## Abstract

**Introduction:**

People from backgrounds that are economically/socially disadvantaged experienced disproportionately high COVID-19 death rates and had lower vaccination rates. Effective outreach strategies for increasing vaccine uptake during the pandemic are not fully known. Among patients receiving care at a Federally Qualified Health Center, we tested whether community engaged digitally-enabled outreach from a trusted clinician messenger increased COVID vaccine uptake.

**Study design, setting, and participants:**

A 3-parallel-arm randomized controlled trial with a hybrid effectiveness-implementation design was conducted among patients ≥ 18 years old on study enrollment during 2021 with 1,650 assigned in 3:10:20 ratio; 2,328 were later selected for two subsequent implementation rounds.

**Interventions:**

From April 13 to June 10, 2021, patients were proactively sent a text-messaging invitation to make an appointment for vaccination as part of the routine practice with a link to frequently asked questions (Arm 1, *n* = 150) with added personalized clinician recommendation alone (Arm 2, *n* = 500) or with enabled 2-way SMS messaging feature (Arm 3, *n* = 1,000). Further implementation used messaging addressing vaccine hesitancy (*n* = 1,323) or adverse reactions to vaccines (*n* = 1,005).

**Main outcomes and measures:**

The primary outcome was the completion of the first SARS-Cov-2 vaccine dose determined at 14, 30 and 90 days after outreach.

**Results:**

Of 1,650 patients in effectiveness Arms, 61% was female. Vaccination rates for Arms 1, 2, and 3, were 6% (*n* = 9), 5.4% (*n* = 27) and 3.3% (*n* = 33) at 14 days, and 11.5% (*n* = 17), 11.6% (*n* = 58), and 8.5% (*n* = 85) at 90 days, respectively, which were similar in pairwise comparisons. At 90 days, vaccination rates were similar across the two implementation rounds (3.9% vs. 3.6%) and were similar to the rate (3.3%) among patients who were not selected for intervention arms or implementation rounds (*n* = 8,671).

**Conclusions:**

Digitally-enabled outreach that included SMS messaging outreach augmented with clinician recommendations did not improve COVID-19 vaccination rates.

**Trial registration:**

This study is registered at ClinicalTrails.gov Identifier: NC-T04952376.

**Supplementary Information:**

The online version contains supplementary material available at 10.1186/s12889-024-18648-x.

## Introduction

Vaccination is essential for containing pandemics but effectiveness in populations depends on reach, acceptability, and uptake. This is particularly important when multiple doses are needed, as is the case with the severe acute respiratory syndrome coronavirus 2 (SARS-CoV-2) pandemic. Equitable access to coronavirus disease 2019 (COVID-19) vaccination is a national health priority. Effective and scalable strategies for promoting vaccine uptake among people from groups that are economically or socially disadvantaged are essential for addressing COVID health disparities. People from economically or socially disadvantaged groups have lower uptake despite incurring higher hospitalization and mortality rates [1–3]. Primary care clinicians/providers (PCP) are viewed as trusted sources for health information with the potential to promote vaccination [4–6], and digital tools are increasingly used in healthcare and have been used extensively during the COVID-19 pandemic. Direct outreach through text-messaging (short message service [SMS]) is commonly used in healthcare for scheduling appointments or notifying patients of healthcare related issues [7], but has had mixed results for promoting vaccination even with added behavioral nudges [8–11]. The effect of framing SMS outreach as a PCP recommendation for improving vaccination, particularly in settings with low levels of engagement [10], is unclear. Federally qualified health centers (FQHC) in the U.S. serve groups that are underserved with health services, including many receiving Medicaid, or in low-socioeconomic status or “essential” jobs such as in healthcare, transportation, information technology, food and agriculture, public works, and others, which have disproportionately more people from lower socioeconomic or racial minority groups [12, 13]. This study investigated whether among people receiving care at a FQHC in a city with high COVID rates, SMS messages that include PCP endorsement increased COVID-19 vaccination uptake.

## Methods

### Study design

We performed a 3-parallel-arm randomized pragmatic trial to test the effectiveness of proactive population-based outreach during the initial round of vaccinations. Later, two separate rounds of implementation with refined messaging were performed. The study was conducted as part of the Arizona Community Engagement Alliance (CEAL) Against COVID Disparities Community Task Force, which worked to address misinformation, increase trust in vaccination, and identify and address barriers to COVID-19 preventive services during the pandemic [14]. Our approach adapted ongoing strategies at a FQHC that included a survey as part of the CEAL initiative that included items on perceptions about SARS-Cov-2 vaccination. We collaborated with the leadership of the FQHC, which participates in the Arizona CEAL Community Taskforce. The study was registered on clinicaltrials.gov (NCT04952376) and approved by the Mayo Clinic IRB (Protocol # 21-002939), which waived informed consent requirements.

### Study population

**Fig. 1:**
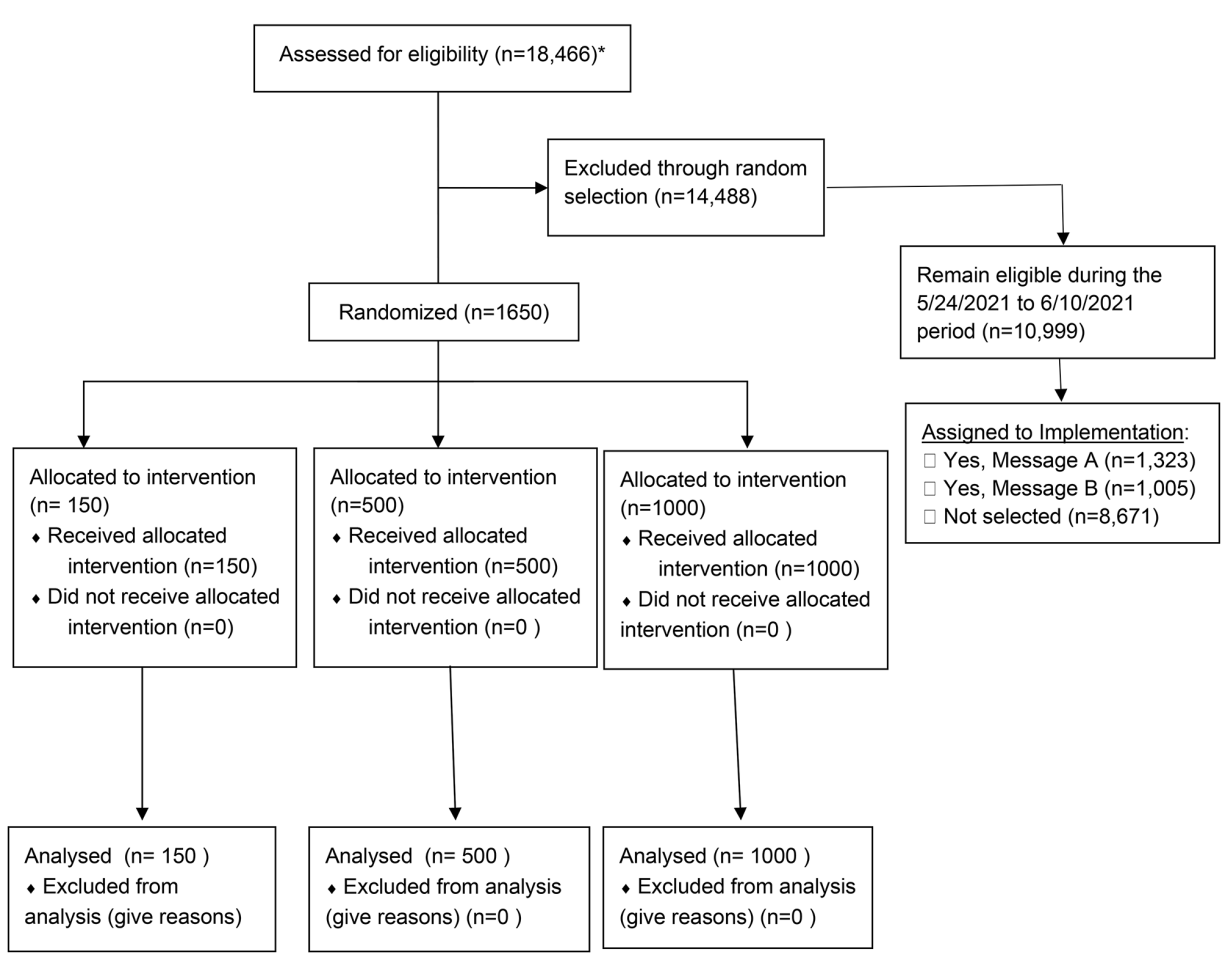
CONSORT flow diagram: *Note: We identified patients who during the 3/30/2020 to 3/29/2021 period were 18 years or older and had a medical visit. Visits were not limited by the type of clinician, clinic location, or vaccine status. We then restricted to those who had at least one visit with an MD or DO (FM or IM providers ONLY) during that period and did not have: •Upcoming scheduled vaccine appointment •prior COVID vaccine dose documented in the electronic health record Patients whose last visit was with an FNP or PA or whose visit occurred in two specific clinical locations (Wickenburg and Gila Bend) were excluded

The study was conducted among people ≥ 18 years old who received care between April 13 to June 10, 2021 at Adelante Healthcare, which is a FQHC with community-based ambulatory primary care clinics in Phoenix, Arizona. FQHCs provide comprehensive primary care services regardless of insurance status or ability to pay [13]. Eligible patients were identified using the electronic health record (EHR). We excluded people with documented vaccination; no medical visit within the previous year; and an upcoming appointment within 1 month (Fig. 1). Among those eligible (*n* = 18,466), we randomly selected and assigned 1,650 patients to one of 3 effectiveness arms in a 3:10:20 ratio per protocol.

### Interventions

The intervention was built on an existing population outreach program at Adelante FQHC that is implemented in partnership with Providertech, a healthcare technology company that operates an outreach platform with automated workflows. Because of observed trends of declining vaccination rates at Adelante and the region at the time the intervention was implemented (Supplementary Materials Table S1) and in consideration of CEAL’s interest in equitable access and to increase likelihood of engagement, we enabled the ability to respond via SMS and included a link to frequently asked questions (FAQs) for all participants (Supplementary Materials Table S2). Thus, all participants, irrespective of the study arm, could reply to the text message, and schedule an appointment or speak with clinical staff through a dedicated phone number provided through the Providertech platform (Supplementary Materials Figure S1).

People assigned to Arm 1 (*n* = 150) received the usual practice of proactive SMS outreach to the cell phone on file with a message that “it is your turn” to get vaccinated, an invitation to make a vaccination appointment and a link in the messaging to FAQs (Supplementary Materials Table S2). In Arm 2 (*n* = 500), the message was personalized by including a statement from the PCP recommending vaccination. Arm 3 (*n* = 1,000) was similar to Arm 2 and a 2-way SMS feature was enabled for 2-way dialogue with the clinical team.

FAQs were hosted on a cloned Adelante website that was created for the purposes of the study. Messages were developed through a community-engaged process and delivered in English and Spanish, based on patient preferred language on file, at preset times for each patient each week with up to 3 reminders. We used the SMS-capable device on file and the technology platform can record undelivered messages.

The health center subsequently tested two implementation alternative messaging, which were sent separately on May 24, 2021, and 2 weeks later (June 10, 2021), based on responses to the preliminary survey. For this evaluation, we considered 10,999 patients who remained unvaccinated, had not previously been selected for the randomized trial, had a medical visit in the previous year, and did not have an upcoming appointment within 1 month. The messaging outreach addressed potential COVID-19 vaccine hesitancy (Message A, *n* = 1,323) or concerns about “side effects” or immune response to vaccination (Message B, *n* = 1,005).

### Study outcome

The primary outcome was the completion of the first COVID-19 vaccine dose at day 14, 30, and 90 after the outreach messaging as determined from EHR data.

### Statistical analysis

Power calculations were based on data from other states in the CEAL program and had assumed a 42% vaccination rate in Arm 1 with a projected 20% higher rate in response to PCP recommendation and an additional 10% higher uptake in Arm 3 than Arm 2. Sample size was therefore estimated as 156 per arm for Arms 1 vs. 2 comparisons and 552 per arm for Arms 2 vs. 3 comparisons for 80% power at a 2-sided Bonferroni-corrected alpha of 0.017. We allowed for the potential for 20% ineligibility after randomization.

We used 2 × 2 contingency tables with the Chi-square test to perform pairwise comparisons of vaccination rates among the three Arms at each time point using an intention-to-vaccinate analysis. Similar analyses were performed between implementation rounds (*n* = 2,328) and with comparisons to people not included in intervention or implementation (*n* = 8,671). Analyses were performed in STATA (StataCorp. 2019. *Stata Statistical Software: Release 16*. College Station, TX). Consolidated Standards of Reporting Clinical trial (CONSORT checklist) was followed to report the data.

## Results

At time of randomization, a total of 18,466 eligible patients were evaluated for the study, of whom 1,650 were randomly assigned to one of the 3 Arms (Fig. 1). Of those, 61.1% (*n* = 1,008) were female, 64.4% (*n* = 1,063) was White, 7.9% (*n* = 130) Black or African American, and 47.4% (*n* = 782) Hispanic/Latino that varied across Arms (Table 1).

Across the 3 arms, 88 patients reported prior vaccination. In an intention to vaccinate analysis, at 2 weeks of follow-up, the vaccination rates in Arms 1, 2, and 3 were 6% (*n* = 9), 5.4% (*n* = 27) and 3.3% (*n* = 33), respectively. The difference was not statistically significant (*P* = 0.78 to 0.05). Vaccination rates at 90 days (11.3% (*n* = 17), 11.6% (*n* = 58), and 8.5% (*n* = 85), respectively, for Arms 1, 2, and 3, which remained similar across groups in pairwise comparisons (*P* = 0.07 to 0.05).

In addition to the randomized trial, we evaluated two rounds of outreach by assigning 2,328 patients to one of two messages based on survey results, and 8,671 were not selected for outreach messaging. Among the 2,328 patients who received outreach messaging, 650 (27.8%) responded back using the two-way platform. When the vaccination rates of the two implementation rounds were assessed at 90 days, both rounds had lower response rates than Arm 3 (*p*-value < 0.01), and response rates to outreach addressing reactions or side effects or hesitancy were similar (Table 2). Among that 8,671 patients at Adelante who were not selected for the interventions, vaccination rates were 0.44%, 1.3% and 3.3% at 14, 30, and 90 days, respectively. We performed sensitivity analyses using data from EHR and population registries, including self-reported information, to exclude patients who reported prior vaccinations, which did not change the findings.

**Table 1:** Characteristics by Intervention Group Assignment of the Trial (*N* = 1,650)

Characteristic	Arm 1 (*n* = 150) n (%)	Arm 2 (*n* = 500) n (%)	Arm 3 (*n* = 1000) n (%)
**Female**	92 (61.3)	323 (64.6)	593 (59.3)
**Age (years)**			
18–29	26 (17.4)	80 (16.0)	172 (17.2)
30–39	26 (17.3)	89 (17.8)	186 (18.6)
40–49	31 (20.7)	118 (23.6)	238 (23.8)
50–59	29 (19.3)	94 (18.8)	166 (16.6)
60–69	27 (16.9)	81 (16.2)	163 (16.3)
70+	11 (7.3)	27 (7.6)	53 (7.5)
**Race**			
White or European American	82 (54.7)	323 (64.6)	658 (65.8)
Asian American	3 (2.0)	15 (3.0)	38 (3.8)
Black or African American	12 (8.0)	40 (8.0)	78 (7.8)
American Indian/Alaskan Native	4 (2.7)	4 (0.8)	6 (0.6)
Native Hawaiian and other Pacific Islanders	0	2 (0.4)	9 (0.9)
More than one race	4 (2.7)	5 (1.0)	15 (1.5)
Unknown	45 (30.0)	111 (22.0)	196 (19.6)
**Ethnicity**			
Hispanic/Latino	82 (54.7)	249 (49.8)	451 (45.1)
Not Hispanic/Latino	62 (41.3)	231 (46.2)	522 (52.2)
Unknown	6 (4.0)	20 (4.0)	27 (2.7)

**Table 2:** Outcomes of study participants according to trial arms (A: *n* = 1,650), implementation waves (B: *N* = 2,328) and those not selected for implementation (*n* = 8,671)

A: Effectiveness Trial Results			
	Arm 1 N = 150, n (%)	Arm 2 N = 500, n (%)	Arm 3 N = 1000, n (%)
14-day	9 (6.0)	27 (5.4)	33 (3.3) ^¶^
30-day	14 (9.3)	42 (8.4)	49 (4.9) *^¶^
90-day	17 (11.3)	58 (11.6)	85 (8.5)

B: Implementation Phase Study			
	Round 1 N = 1,323, n (%)	Round 2 N = 1,005, n (%)	Not Selected for Outreach N = 8,671, n (%)
14-day	12 (0.9)	6 (0.6)	44 (0.5)
30-day	22 (1.7)	14 (0.6)	89 (1.0) ^‡^
90-day	51 (3.9)	36 (3.6)	286 (3.3)

**Significance**:

1vs. 3; *p < 0.05

2 vs. 3: ¶ *p* < 0.01

Baseline vs. Round 1: ‡ *p* < 0.05

## Discussion

Due to its simplicity, low implementation cost, and scalability, digitally enabled outreach is an attractive approach for raising awareness on emerging public health issues and clinicians are considered trusted sources of information. In this study of a proactive population outreach intervention, adding a clinician recommendation did not improve the effectiveness of SMS messaging in improving COVID-19 vaccine uptake. Refining the strategy by offering to address concerns about vaccination also did not increase uptake. Our findings are in line with studies of proactive outreach in settings with low immunization rates that also reported similarly low uptake in response to an intervention such as a 3.1–3.6% response rates to SMS +/- telephone calls [10]. Our study was unique in the use of community-engaged approaches in a low-resource setting. The underlying rates of vaccination during the study period were low at the FQHC, reflecting waning interest in the community over time in 2021 [15]. Consistent with our results, in previous studies of COVID and influenza vaccines that reported increased uptake with SMS behavioral nudges, the increases were modest [8, 9]. The ongoing use of SMS messaging in the population may have reduced the effectiveness of the intervention or nudges in our studies and may explain the lower uptake in Arm 3 and during implementation rounds. Thus, our results may be due to low engagement in vaccination related to sociodemographic factors, hesitancy, distrust, misinformation, and other barriers [5], which may not be easy to overcome with trusted messengers through SMS digital media.

### Limitations

The benefit of digital tools during the pandemic is understudied in FQHCs. A limitation of SMS is the limited ability to deliver robust structured education or motivational counseling content that may be needed to address hesitancy. Our incorporation of interactive components did not improve effectiveness. There are other limitations of our study, including uncertainties about the completeness of capture of vaccinations received outside the health center, but findings were unchanged in sensitivity analyses. The digital divide may have also played a role in engagement in the intervention, but we were unable to assess the impact of digital health inequity on the response to SMS. Phone calls and other forms of outreach for vaccinations are necessary for patients who have limited access to emails or internet services [11]. We could not verify patient contact information before executing outreach. Community engagement is believed to promote vaccine uptake, and the CEAL programs had active community-wide vaccine education outreach during the time of the study [14]. However, the effectiveness and reach of such strategies could not be assessed in this study. It is possible for patients to have a stronger relationship with a clinician other than the one used in SMS, which may diminish the effectiveness of the intervention, but such influence is expected to be similar across arms.

## Conclusions

Our results suggest that adding personalized physician recommendation to SMS messaging and a two-way interactive feature offering information about availability and addressing concerns is a feasible form of COVID vaccine outreach in under-resourced communities but did not increase uptake. Further research is needed on effective technology-enabled outreach strategies in populations receiving care in FQHCs.

### Electronic supplementary material

Below is the link to the electronic supplementary material.

Supplementary Material 1

## Data Availability

The data that support the findings of this study are available from Adelante Healthcare, but restrictions apply to the availability of these data, which were used under license for the current study, and so are not publicly available. Data requests can be sent to Chyke A. Doubeni MD, MPH at chyke.doubeni@osumc.edu.

## Declarations

This study adheres to the principles of the Declaration of Helsinki.
