# Supplementary material for: The effect of adding physician recommendation in digitally-enabled outreach for COVID-19 vaccination in socially/economically disadvantaged populations
Source: BMC Public Health. 2024 Jul 18;24:1933. doi: 10.1186/s12889-024-18648-x (PMC11264853; doi:10.1186/s12889-024-18648-x)

Supplementary Materials

The Effect of Adding Physician Recommendation in Digitally-Enabled Outreach for COVID-19 Vaccination in Socially/Economically Disadvantaged Populations

Table S1: COVID-19 Vaccination Rates by Quarter in 2020 and 2021, Adelante Healthcare

|  | 2020 | 2021 | | | |
| --- | --- | --- | --- | --- | --- |
|  | Q4 | Q1 | Q2 | Q3 | Q4 |
| Total Patients Seen | 32,161 | 34,537 | 40,049 | 35,844 | 35,925 |
| 1^st^ Dose | 105 (0.3) | 8,829 (25.6) | 11,170 (27.9) | 5,196 (14.5) | 4,744 (13.2) |
| 2^nd^ Dose | 2 (0) | 3,907 (11.3) | 9,471 (23.6) | 3,447 (9.6) | 1,885 (5.2) |
| 3^rd^ Dose | 0 (0) | 8 (0.0) | 20 (0.0) | 157 (0.4) | 2,783 (7.7) |
| Any dose | 108 (0.3) | 12,744 (36.9) | 20,664 (51.6) | 8,806 (24.6) | 9,423 (26.2) |

**Note**: Rates reported reflect vaccines administered at the clinic and self-reported by patients and recorded in the electronic health record at the time of the intervention and may not accurately reflect vaccination received elsewhere

Table S2: Content and Sequence Messaging Used in the Trial and Implementation

| Protocol | Message Content | Invitation with link to FAQs | PCP recommends | 2-way SMS | Date(s) Sent |
| --- | --- | --- | --- | --- | --- |
| Arm 1 | Hi [FirstName] it is your turn to get a COVID-19 vaccine. Please call Adelante for an appt 602-601-3050. Questions about the vaccine? Simply reply to ask 5=STOPP <URL to FAQ landing page> | √ |  |  | 4/13/2021 |
| Arm 2 | Hello [FirstName], it's [ProviderName] at Adelante. It is your turn for a COVID-19 vaccine. I recommend that you call 602-601-3050 for an appt. Questions? Simply reply to this message. 5=STOP <URL to FAQ landing page> | √ | √ |  | 4/13/2021 |
| Arm 3 | Hello [FirstName], it's [ProviderName] at Adelante. It is your turn for a COVID-19 vaccine. I recommend that you call 602-601-3050 for an appt. Questions? Simply reply to this message. 5=STOP <URL to FAQ landing page> | √ | √ | √ | 4/26/21, 4/27/21 |
| Implementation to address addressing concerns | Concerns about getting the vaccine [FirstName]? [ProviderName] from Adelante can help answer your questions. Text us or call 602.601.3050. 5=STOP <URL to FAQ landing page> |  |  | √ | 5/24/2021, 6/10/21 |
| Implementation to address potential reaction | Concerns about your response to the vaccine [FirstName]? [ProviderName] from Adelante can help answer your questions. Text us or call 602.601.30505=STOP <URL to FAQ landing page> |  |  | √ | 5/24/2021, 6/10/21 |

Notes: FAQs = Frequently asked questions adapted from the Centers for Disease Control and Prevention and from community input; PCP = Primary care clinician/provider; URL=Uniform Resource Locator (the address for the cloned Adelante website)

Figure S1: ProviderTech Vaccine Communication Workflow


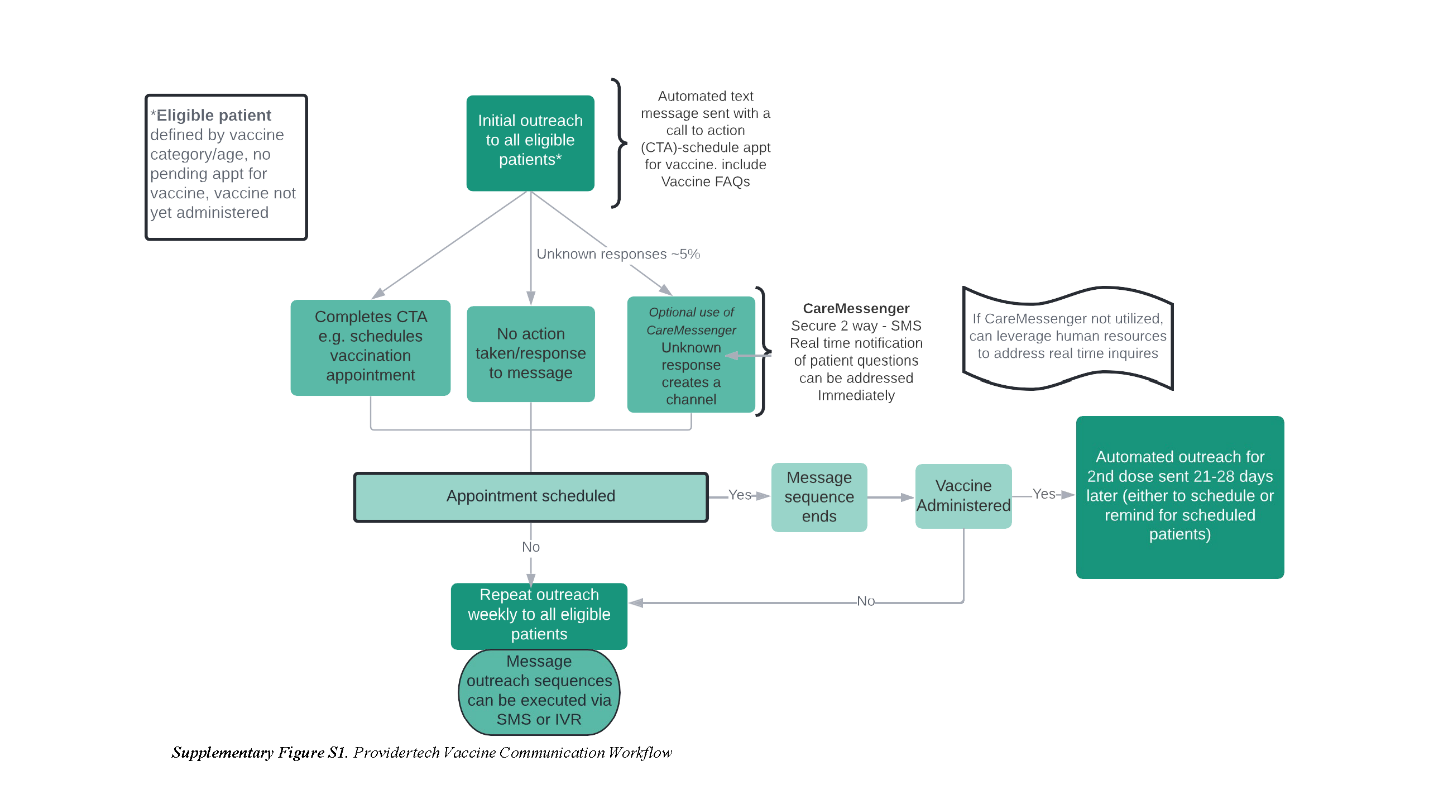

Supplement: Supplementary file 1 — Supplementary Material 1 [file 12889_2024_18648_MOESM1_ESM.docx]
